# The Landscape of Circulating miRNAs in the Post-Genomic Era

**DOI:** 10.3390/genes13010094

**Published:** 2021-12-30

**Authors:** Fabio Lauria, Giuseppe Iacomino

**Affiliations:** Institute of Food Sciences, National Research Council, Via Roma, 64, 83100 Avellino, Italy; flauria@isa.cnr.it

**Keywords:** microRNA, circulating miRNA, gene expression, theranostic biomarkers

In the past decade, there has been an epochal change in the way that diseases are investigated and diagnosed. The advent of high-throughput technologies has placed us firmly into the post-genomic era. Omics investigation techniques have advanced quickly in the field of personalized medicine, exerting great influence on the progress of epigenetics research [1]. In essence, every step in gene expression flow is finely controlled, and the discovery of small non-coding RNAs (sncRNAs) has added new contributors to the already well-specialised supervisory mechanisms.

Based on their methods of operation, assembly, and structure, sncRNAs can be classified as small interfering RNAs, PIWI-interacting RNAs, endogenous small interfering RNAs, promoter-associated RNAs, small nucleolar RNAs, and microRNAs (miRNAs). These last molecules are sncRNAs (20–24 nucleotides in length), which act as post-transcriptional regulators of gene expression. Up until now, 2599 different human miRNAs have been identified and deposited in the miRTarBase [2]. Since the first discovery of a noncoding RNA in *C. elegans* in 1993 [3], the knowledge of miRNAs has progressed, with thousands of papers clearly defining their crucial role in a wide range of disorders and physiological processes. These molecules typically act as specific gene silencers by base-pairing to the 3′ untranslated sequence of a target mRNA, but they have also been reported to bind anywhere along the mRNA sequence. Interestingly, miRNAs work by either suppressing translation or by affecting the stability and degradation of the target mRNA. The nucleotides in positions 2–8 of a mature miRNA have been designated as the “seed sequence” because they are required for base pairing with a target mRNA. Moreover, non-canonical seed-like consensus sequences also mediate a portion of miRNA-target recognition. Sequence identity in the seed region has also been employed to group miRNAs into “families” that share the common ability to recognize clusters of target mRNAs. While a limited number of miRNAs have a typical tissue-specific localization, the majority of them exhibit broad tissue distribution.

A certain miRNA can target a set of transcripts at the same time; moreover, a single mRNA typically includes numerous interaction sites for different miRNAs, thus generating complex regulatory circuits. Even though a specific miRNA commonly exerts limited activity on a particular target, its activity, which influences multiple transcripts in a signalling network, leads to substantial cumulative effects. As a result, Metazoan miRNAs have been denoted as the “sculptors” of the cell transcriptome [4], and in agreement with scientific evidence, endogenous miRNAs are capable of affecting the expression of up to 40–60% of mouse and human genes. However, with millions of hypothetically conceivable miRNA–mRNA interactions, the human miRNAs targetome is far from being determined [5].

Given the ubiquity of nucleases, traditional views suggest that RNAs are not stable molecules in extracellular settings. Conversely, in the recent past, several miRNAs have been shown to retain unexpectedly high concentrations of plasma and other bodily fluids. Circulating miRNAs (c-miRNAs) are not naked molecules and are commonly secreted from cells that are arranged into complexes with proteins, microvesicles, or exosomes. Interestingly, extracellular miRNAs can be actively secreted and transferred into recipient cells where they may influence the translation of the target gene/s, playing a role in intercellular communication [6]. Despite this, most of the functional significance of c-miRNAs is unknown.

The discovery and validation of biomarkers in prognosis, diagnosis, drug discovery, treatment, and prevention play a key role in the post-genomic era. Therefore, the prognostic and diagnostic potential of c-miRNAs as non-invasive biomarkers has been advocated in the health of both humans and animals [7,8]. In this regard, the identification of about 300 extracellular miRNAs in several biological fluids has emphasized their potential in biomarker development in next-generation medicine [7]. Accordingly, the dysregulation of miRNAs has been established to reflect the status and functions of various tissues and organs, most likely contributing to their anomalies [9]. In this context, many studies support the links between miRNAs and the physiopathology of a variety of processes, including mitochondrial failure, cardiovascular diseases, neurodegenerative disorders, cancer, and other conditions, underlining their relevance for personalized medicine applications. Moreover, the role of miRNAs in maintaining an energy balance and metabolic equilibrium in living organisms, which is achieved by regulating different metabolic pathways, has been established [10]. Likewise, several differentially expressed miRNAs have been characterized in the treatment of infectious diseases [11]. Several studies have also recognized that miRNA expression is altered in response to nutrition and lifestyle variables [12], and numerous microRNA families have been interconnected to different dietary regimes. Accordingly, the recent nutrimiRomics discipline was inspired by the influence of diet on miRNA levels and the sub-sequential effects on gene expression and health-related conditions. Increasing evidence also suggests that dietary miRNAs may survive digestion [13]; nevertheless, the role and capability of food-related miRNAs to modulate cross-species messengers remain puzzling.

Of note, the majority of the 139,968 papers that have been written on miRNAs research that are listed in PubMed (November 2021) show their implications in human diseases, emphasizing that miRNAs are highly selective health-related tools [14], with the bulk of these studies focusing on c-miRNAs as theranostic cancer biomarkers [15,16,17]. However, the number and variety of the studies reveal the extraordinary complexity of the topic, and, at present, there is still a lack of consensus over specific miRNA profiles that are useful for the early identification of cancer cells in vivo. Most of the working limits are related to approaches that would be useful for c-miRNA recognition, which must be strictly specific and capable of identifying limited amounts of target molecules. Given the conceivable presence of unwanted contaminant inhibitors, these procedures should be strictly specific and capable of identifying limited amounts of target molecules. The inconsistencies among studies are correlated, at least in part, to dissimilarities in the extraction and identification procedures, in the experimental design, and data normalization, with these limitations supporting the need for the development of well-standardized protocols and operational strategies [18,19].

Based on technological and conceptual progress in the field of personalized medicine, new therapeutic opportunities are presented in clinical medicine by molecular drugs that have been inspired by the target sequence. In this context, recent trials have predicted that both anti-miR and miR-mimics compounds can be used as drugs for various therapeutic applications, and numerous biopharmaceutical companies are now involved in this business [20]. However, caution is be advised based on lessons that have been learned in the past by the pioneering trials with antisense oligonucleotides. These synthetic molecules, which act as specific gene silencers by base-pairing to the target RNA, have only offered a limited number of therapies in clinical applications despite expectations and after 30 years of research [21]. Accordingly, the development of miRNA-based therapeutics is in its infancy, and many concerns remain to be resolved, including appropriate delivery systems, stability, bioavailability, etc. [22].

This Special Issue aims to provide a broad overview of the recent knowledge on several open questions and new opportunities in the field of miRNA research and the use of miRNAs as early diagnostic tools, providing evidence of the effectiveness and implications of this class of small molecules that plays a big role in gene expression control.
